# Hydroxychloroquine blood concentrations and effects in Chinese patients with IgA nephropathy

**DOI:** 10.1007/s40620-024-02029-z

**Published:** 2024-07-24

**Authors:** Ting Yang, Yaotong Shi, Ye Wang, Yuan Feng, Qiuyuan Shao, Chunming Jiang, Lulu Wang, Jing Liu

**Affiliations:** 1 Department of Nephrology, Affiliated Hospital of Medical School, Nanjing Drum Tower Hospital, Nanjing University, Nanjing, China; 2 Department of Pharmacy, Affiliated Hospital of Medical School, Nanjing Drum Tower Hospital, Nanjing University, Nanjing, China; 3 Department of Nephrology, Nanjing Drum Tower Hospital Clinical College of Jiangsu University, Zhenjiang, China; 4 Department of Pharmacy, Nanjing Brain Hospital, Nanjing, China

**Keywords:** IgA nephropathy, Hydroxychloroquine, Blood concentration, Proteinuria

## Abstract

**Background:**

Hydroxychloroquine (HCQ) is recommended for Chinese patients with immunoglobulin A nephropathy (IgAN). However, the relationship between HCQ blood concentration and the therapeutic effect for IgAN has not yet been defined. This study investigates the optimal and efficacious range of HCQ blood concentrations in Chinese patients with IgAN.

**Methods:**

Seventy-three patients with biopsy-proven IgAN who were at risk of progression were included in this study. Thirty-eight patients with IgAN were treated with HCQ plus an optimized renin–angiotensin–aldosterone system inhibitor (RAASi), and thirty-five patients received only RAASi. Blood HCQ concentration and 24-h proteinuria were examined at three and six months after treatment.

**Results:**

The baseline proteinuria levels were comparable between the RAASi and HCQ groups. The HCQ group had lower 24-h proteinuria than the RAASi group three months after treatment, though the difference was not significant (*p* = 0.38). After six months, the median proteinuria level was significantly lower in the HCQ group than in the RAASi group (*p* < 0.05). The percentage reduction in 24-h proteinuria in the HCQ group was greater than that in the RAASi group at three (*p* < 0.05) and six months (*p* < 0.05). Hydroxychlorquine blood concentration and efficacy were positively correlated at three months (*r* = 0.428, *p* < 0.05) and six months (*r* = 0.48, *p* < 0.05). Moreover, the optimal blood concentration of HCQ for three-month efficacy was 418.96 ng/mL and that for six-month efficacy was 582.48 ng/mL. No serious adverse events were reported during HCQ treatment.

**Conclusions:**

Hydroxyhloroquine safely reduces proteinuria in Chinese patients with IgAN. The efficacy of HCQ is positively correlated with its blood concentration.

**Graphical abstract:**

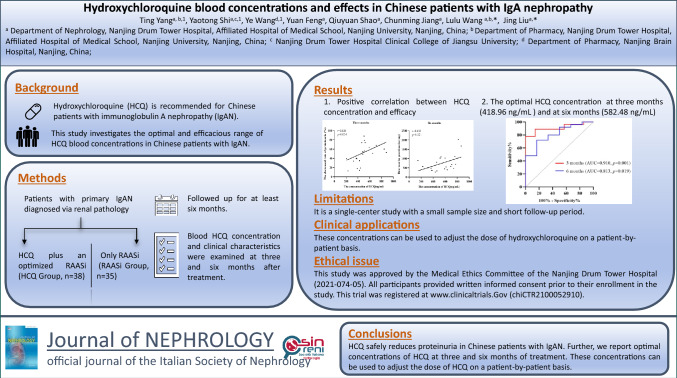

**Supplementary Information:**

The online version contains supplementary material available at 10.1007/s40620-024-02029-z.

## Introduction

Immunoglobulin A nephropathy (IgAN) is the most common form of primary glomerulonephritis in the world and is also an important cause of end-stage kidney disease (ESKD) [1]. Due to the uncertainty of its pathogenesis, there are no specific treatment options for IgAN. The treatment paradigm for IgAN promoted by Kidney Disease: Improving Global Outcomes (KDIGO) is optimal supportive care, including blood pressure management, optimal tolerated doses of renin–angiotensin–aldosterone system inhibitors (RAASi), and lifestyle modifications. Patients at high risk of chronic kidney disease (CKD) progression after three months of optimal therapy may be considered for corticosteroid therapy; however, the benefits and risks should be weighed in the context of the individual patient [2]. Furthermore, there is controversy and uncertainty regarding the efficacy of corticosteroids and immunosuppressants for the management of IgAN [3]. In recent years, clinicians have focused on new drugs for patients with IgAN based on increasing research regarding the pathogenesis of IgAN. Interestingly, hydroxychloroquine (HCQ) is the only new therapeutic strategy recommended for Chinese patients with IgAN in the 2021 edition of the KDIGO [2].

Hydroxychloroquine is widely used as an immunomodulator for autoimmune diseases, including rheumatoid arthritis and systemic lupus erythematosus (SLE) [4, 5]. Several recent studies have demonstrated the potential of HCQ in reducing proteinuria in patients with IgAN. Liu et al*.* reported that HCQ, in combination with an optimized RAASi, decreased proteinuria in Chinese patients with IgAN over a six-month period without adverse events [6]. Tang et al*.* [7] reported decreased proteinuria from 1.69 g/ day to 1.01 g/day at 12 months and to 1.00 g/day at 24 months after HCQ treatment in patients with IgAN with no serious adverse events, though the estimated glomerular filtration rate (eGFR) was not significantly different at 12 months. These results indicate that HCQ is effective and safe for patients with IgAN.

The administered dose of HCQ was based on eGFR in both previous studies; however, HCQ is currently prescribed according to body weight in patients with autoimmune diseases. Several previous studies that evaluated the efficacy of HCQ in patients with SLE administered at least 6.5 mg/kg body weight per day [8]. However, Fasano et al*.* suggested that the risks and benefits of treating SLE must be balanced when choosing the HCQ dose, and that the measurement of HCQ plasma concentration can help clinicians adjust the daily dosage according to individual pharmacokinetic variability [9]. Moreover, Cunha et al*.* reported patients with renal flares have lower mean HCQ levels during follow-up than those in continuous remission and that the likelihood of renal flares was decreased when an HCQ target > 600 ng/mL was applied in patients with SLE [10]. A low blood concentration of HCQ is a marker and predictor of disease exacerbation in patients with SLE [11]. However, specific measurements of the HCQ blood concentration in patients with IgAN have yet to be reported. This study determines the relationship between HCQ blood concentration and its efficacy for patients with IgAN, and reports the HCQ blood concentration at the time of optimal efficacy.

## Methods

### Study population

This single-blind, randomized clinical trial (RCT) included patients with primary IgAN diagnosed via renal pathology who were treated at Nanjing Drum Tower Hospital, Affiliated Hospital of Medical School, Nanjing University from January 2020 to June 2021. All patients were 18–75 years old with a pathological diagnosis of primary IgAN, an eGFR > 30 ml/min/1.73 m^2^ (calculated using the CKD Epidemiology Collaboration creatinine equation [12]), and a proteinuria of 0.75–3.5 g/d despite receiving the maximum dose of RAASi for at least three months. Patients with secondary IgAN, systemic use of corticosteroids or immunosuppressants within three months, specific pathological or clinical renal disease types (such as nephrotic syndrome or crescentic nephritis (pathological diagnosis)), IgAN requiring corticosteroid therapy, or contraindications to HCQ therapy were excluded from the study. Pregnant or breastfeeding patients and those who planned to get pregnant were also excluded from the study, as were patients who were lost to follow-up or had poor adherence to the study treatment. The study was terminated if patients experienced serious adverse events or a decrease in eGFR > 30% during the trial. The inclusion and exclusion criteria are shown in Supplementary Fig. S1. This trial was registered at www.clinicaltrials.Gov (chiCTR2100052910).

### Study design

The patients were divided into RAASi and HCQ groups at a 1:1 ratio using a randomized, single-blind method. The control group was treated with RAASi alone and the HCQ group was treated with RAASi and HCQ. In the HCQ group, the administered HCQ dose varied according to baseline eGFR. For patients with an eGFR > 60 ml/min/1.73 m^2^, 0.2 g HCQ was administered twice daily, and for those with 45 ml/min/1.73 m^2^ < eGFR < 59 ml/min/1.73 m^2^, 0.1 g HCQ was administered three times per day. Patients with 30 ml/min/1.73 m^2^ < eGFR < 44 ml/min/1.73 m^2^ were administered 0.1 g HCQ two times per day [6]. All patients were continuously treated with the maximum tolerated dose of the RAASi, and no other treatment was administered during the study period. Patients were evaluated at baseline, and at three and six months thereafter. Blood and urine samples were collected to detect proteinuria and serum creatinine (Scr), albumin (ALB), blood urea nitrogen (BUN), total cholesterol, triglycerides, low-density lipoprotein cholesterol (LDL), and fasting blood glucose. Whole-blood HCQ concentrations were determined using high-performance liquid chromatography.

### High-performance liquid chromatography

Acetonitrile was added to 100 μL of whole blood sample. After the protein was precipitated, the supernatant was removed via centrifugation. An Agilent 1200 Series high-performance liquid chromatography system was used. The concentrations of HCQ and its metabolites were determined using high-performance liquid chromatography with chloroquine as an internal standard. Method validation involved determination of the linear range, accuracy, precision, and limits of quantification. The detailed procedures and the acceptance criteria used to validate the assay have been described previously.

### Outcomes

The primary outcomes were the changes in proteinuria and Scr levels from baseline to three and six months of treatment in both groups. The secondary outcomes included the changes in proteinuria, serum ALB, BUN, total cholesterol, triglycerides, LDL, and adverse events in the HCQ group from baseline to three and six months after treatment.

Data regarding adverse events were collected from the patients’ medical records. In this study, adverse events included gastrointestinal reactions (such as nausea and vomiting), skin allergies, liver function abnormalities, drug-induced renal function progression, and retinal or corneal damage. Serious adverse events include those resulting in serious disability, life-threatening conditions, new-onset diabetes, severe or fatal infection, osteonecrosis of the femur, bone fracture, cardiocerebral vascular disease, cataracts, or gastrointestinal hemorrhage [13].

### Statistical analysis

Normally-distributed data are presented as mean and standard deviation, and non-normally distributed data are presented as median and interquartile range (IQR). Categorical data are expressed as number and frequency. Student's *t*-tests were used to compare the baseline characteristics between the groups for normally distributed continuous variables, while the Wilcoxon signed-rank test was used for non-normally distributed continuous variables. Chi-square tests were used for nominal variables. The correlation between the HCQ concentration and changes in proteinuria was investigated using Pearson’s correlation. The HCQ blood concentrations at optimal efficacy were inferred using receiver operating characteristic (ROC) curves. All analyses were conducted using SPSS Statistics (IBM, version 24.0). Statistical significance was set at *p* < 0.05.

## Results

### Baseline characteristics

The study included 100 patients. A total of 73 eligible patients were included in the final analyses, including 38 in the HCQ group and 35 in the RAASi group. Patient age, body weight, blood pressure, ALB, BUN, triglycerides, total cholesterol, and LDL concentrations, fasting blood glucose, Oxford histologic score and the proportion of patients with diabetes were not significantly different between the groups. At baseline, 24-h proteinuria (942.50 mg/d [IQR: 685.00–1725.92 mg/d] vs. 832.00 mg/d [IQR: 701.00–1395.00 mg/d]; *p* = 0.6) and Scr concentration (77.08 ± 26.51 μmol/L vs. 82.40 ± 25.17 μmol/L; *p* = 0.2) were also different between the two groups (Supplementary Table 1).

### Primary outcomes

**Fig. 1:**
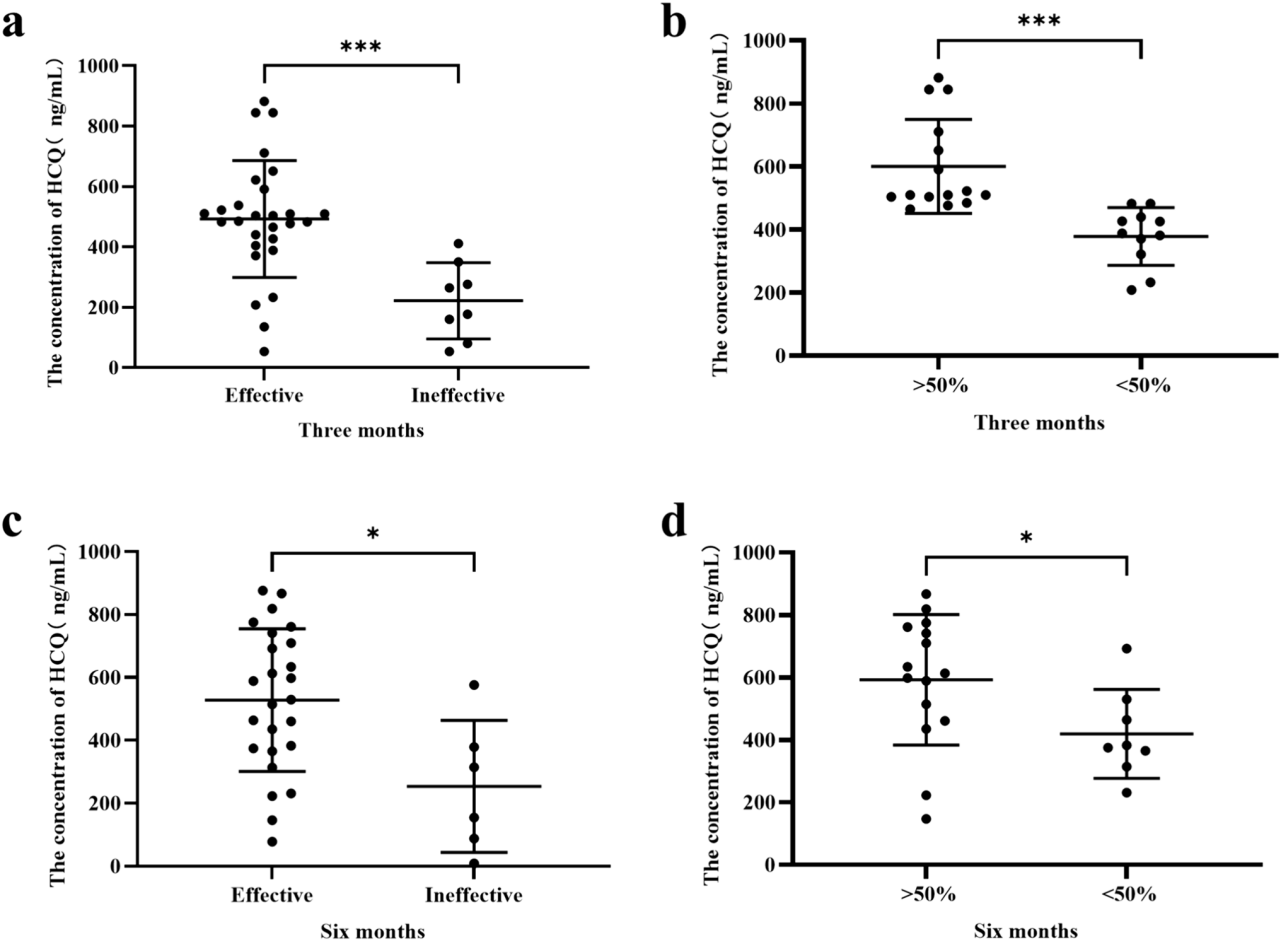
Comparison of HCQ blood concentrations between the effective and ineffective groups. The HCQ blood concentrations were compared between patients in whom HCQ was effective or ineffective at three months (**a**). The HCQ blood concentrations of patients with and without > 50% decrease in proteinuria were also compared at three months (**b**). The same comparisons were made at six months (**c**, **d**). *p < 0.05, ***p < 0.001. *HCQ* hydroxychloroquine

The median 24-h proteinuria in the HCQ group (654.00 mg/d [IQR: 284.00–1161.00 mg/d]) was lower than that in the RAASi group (701.00 mg/d [IQR: 517.00–970.00 mg/d]) after three months of treatment (*p* = 0.38). After six months of treatment, the median 24-h proteinuria was significantly lower in the HCQ group (542.00 mg/d [IQR: 355.00–815.54 mg/d]) than in the RAASi group (883.50 mg/d [IQR: 498.50–1001.20 mg/d]) (*p* = 0.007). Furthermore, the percentage reduction was greater in the HCQ group at three (− 49.23% [IQR: − 66.71 to 13.06%] vs. 5.30% [IQR: − 31.23 to 24.68%]; *p* = 0.004) and six (− 57.50 [IQR: − 68.93 to − 33.63%] vs. − 10.40% [IQR: − 36.32 to 56.45%]; *p* = 0.002) months of treatment. In addition, the proportion of patients whose 24-h proteinuria decreased by more than 50% was higher in the HCQ group than in the RAASi group at three (16/36 vs. 4/31; *p* = 0.006) and six (18/31 vs. 6/30;* p* = 0.002) months of treatment. The Scr concentration was not significantly different between the HCQ and RAASi groups at three (85.14 ± 33.21 μmol/L vs. 79.50 ± 24.71 μmol/L; *p* = 0.45) and six (81.84 ± 26.57 μmol/L vs. 80.74 ± 22.08 μmol/L; *p* = 0.86) months (Table 1). The patients who received HCQ were further divided into two groups based on the effectiveness of the treatment in reducing proteinuria from baseline. The HCQ blood concentrations were higher in the effective group than in the ineffective group at both three (Fig. 1a) and six months (Fig. 1c). The percentage of proteinuria reduction was associated with the HCQ blood concentration. Patients with > 50% reduction of the 24-h proteinuria had higher HCQ concentrations than those with lower reductions (Fig. 1b, d).

**Table 1:** Comparison of primary outcomes between HCQ and RAASi groups at 3 and 6 months

Primary outcomes	Duration of therapy	RAASi group	HCQ group	*p*-value
24-h proteinuria (mg/d)	3 months	701.00 [517.00, 970.00]	654.00 [284.00, 1161.00]	0.38
	6 months	883.50 [498.50, 1001.20]	542.00 [355.00, 815.54]	0.007*
Percent changes in 24-h proteinuria (%)	3 months	5.30% [− 31.23%, 24.68%]	− 49.23% [− 66.71%, 13.06%]	0.004*
	6 months	− 10.40% [− 36.32%, 56.45%]	− 57.50% [− 68.93%, − 33.63%]	0.002*
24-h proteinuria decreased by > 50%	3 months	4 (13%)	16 (44%)	0.006*
	6 months	6 (20%)	18 (58%)	0.002*
Scr (μmol/L)	3 months	79.50 ± 24.71	85.14 ± 33.21	0.45
	6 months	80.74 ± 22.08	81.84 ± 26.57	0.86

*HCQ* Hydroxychloroquine; *RAASi* renin–angiotensin–aldosterone system inhibitor; *Scr* serum creatinine

*Represents statistical significance (*p* < 0.05)

**Fig. 2:**
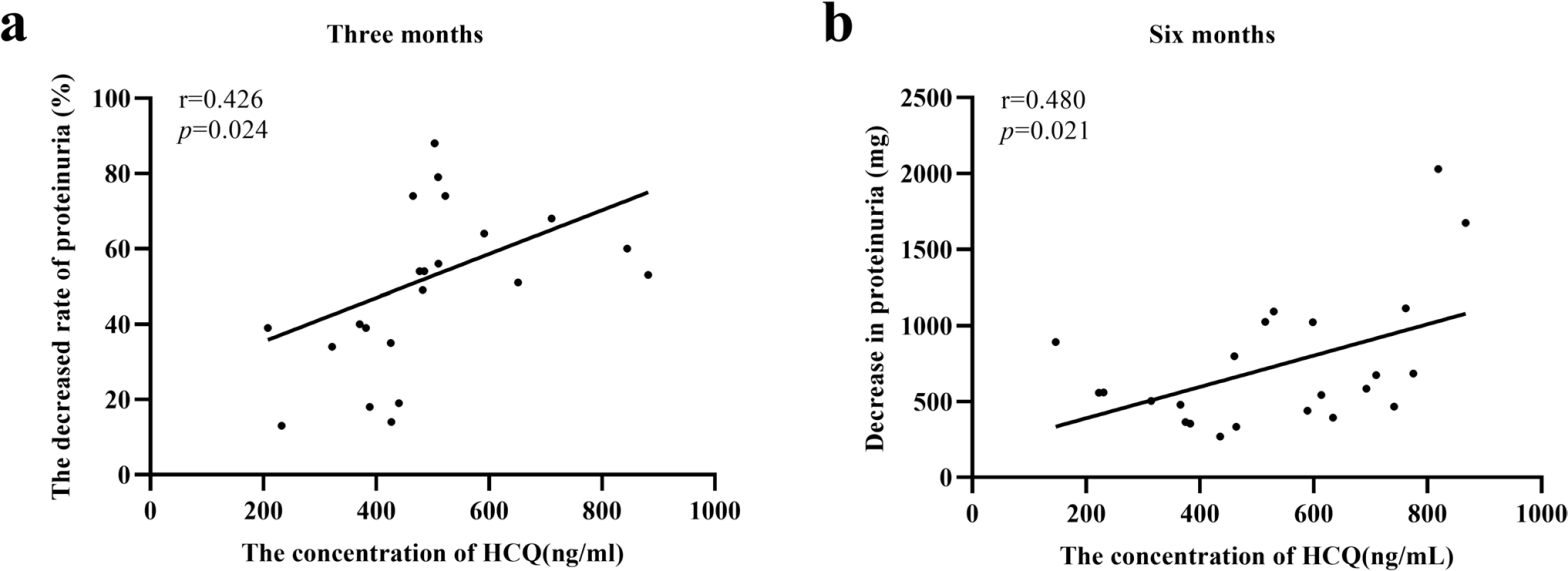
Correlation between HCQ blood concentration and efficacy. **a** The HCQ blood concentration and rate of change in proteinuria at three months are positively correlated (*r* = 0.426, *p* = 0.024). **b** The HCQ blood concentrations and rate of change in proteinuria at six months are positively correlated (*r* = 0.480, *p* = 0.021). *HCQ* hydroxychloroquine

A linear correlation between changes in proteinuria and HCQ blood concentrations at three and six months of treatment was identified. The rate of decrease in 24-h proteinuria at three (*r* = 0.426, *p* = 0.024) (Fig. 2a) and six (*r* = 0.480, *p* = 0.021) (Fig. 2b) months was positively correlated with the HCQ blood concentration.

**Fig. 3:**
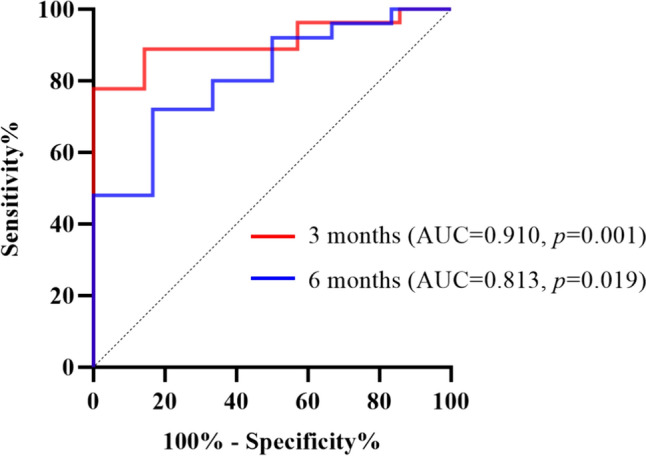
Receiver operating characteristic (ROC) curves. ROC curves reveal the optimal HCQ blood concentrations at three and six months. The sensitivity and specificity of the decrease in proteinuria for different HCQ blood concentrations are shown. *HCQ* hydroxychloroquine

The area under the curve (AUC) for the use of HCQ blood concentration to determine efficacy was 0.91 ± 0.05 (95% CI 0.81–1.00; *p* = 0.001) at three months and 0.81 ± 0.09 (95% CI 0.64–0.99; *p* = 0.019) at six months. The HCQ blood concentration data had a sensitivity of 78% and a specificity of 100% for predicting the optimal efficacy at a cut-off value of 418.96 ng/mL during the three-month follow-up period, and a sensitivity of 48% and specificity of 100% at a cut-off value of 582.48 ng/mL during the six-month follow-up period (Fig. 3).

### Secondary outcomes

Among 31 patients in the HCQ group, the 24-h proteinuria level (727.00 mg/d [IQR: 386.00–983.00 mg/d] vs. 1102.00 mg/d [IQR: 717.00–2081.00 mg/d]; *p* < 0.01), ALB concentration (43.28 ± 2.89 g/L vs. 39.60 ± 3.58 g/L; *p* < 0.001), and LDL concentration (2.41 mmol/L ± 0.66 vs. 2.92 ± 0.93 mmol/L; *p* < 0.05) were significantly different at baseline and three months. The 24-h proteinuria level (542.00 mg/d [IQR: 355.00–815.54 mg/d] vs. 1102.00 mg/d [IQR: 717.00–2081.00 mg/d]; *p* < 0.001) and ALB concentration (43.94 ± 2.53 g/L vs. 39.60 ± 3.58 g/L; *p* < 0.001) were significantly different between baseline and six months. The LDL concentration (2.47 ± 0.99 mmol/L vs. 2.92 ± 0.93 mmol/L; *p* = 0.073) was not different between baseline and six months. The eGFR was not significantly different between baseline and three (98.99 ± 27.96 ml/min/1.73 m^2^ vs. 100.60 ± 26.19 ml/min/1.73 m^2^; *p* = 0.816) or six (95.79 ± 27.07 ml/min/1.73 m^2^ vs. 100.60 ± 26.19 ml/min/1.73 m^2^; *p* = 0.48) months (Supplementary Fig. S2).

### Serious adverse events

No serious adverse events were reported during this study.

## Discussion

The results of this single-blind randomized controlled trial suggest that HCQ plus supportive therapy is safe and effective for reducing proteinuria in patients with IgAN. These results are consistent with those of previous studies [6, 14]. The relationship between HCQ blood concentrations and efficacy was also investigated in this study.

A relationship between HCQ blood concentration and clinical efficacy has been reported in patients with autoimmune diseases [15]. Durcan et al*.* demonstrated that high disease activity in patients with SLE is associated with low HCQ concentrations and that the threshold for clinical efficacy is an HCQ blood concentration > 500 ng/mL [16]. However, the relationship between HCQ blood concentrations and IgAN has not yet been reported. In this study, the HCQ blood concentrations were higher in patients with decreased proteinuria and in patients with > 50% reduction in 24-h proteinuria. After three months of treatment, the percentage of decrease in 24-h proteinuria was positively correlated with HCQ blood concentrations, and the optimal HCQ blood concentration to achieve efficacy at three months was 418.96 ng/mL. The results were similar after six months of treatment with an optimal HCQ blood concentration of 582.48 ng/mL.

A previous clinical trial reported that target-level HCQ doses do not necessarily achieve the therapeutic effect in patients with SLE [17]. In the current study, the patients were administered HCQ based on the eGFR values, with the maximum daily dose set at 400 mg. During six months of treatment, six patients had consistently low HCQ blood concentrations, with a mean value of 43 ng/mL. These patients also had poor treatment outcomes. It is not clear whether this is related to reduced absorption or increased metabolism. Previous studies have confirmed significant individual differences in HCQ pharmacokinetics, and that the 2D6, 3A4, 3A5, and 2C8 isoforms of the cytochrome P450 (CYP) family are likely involved in the metabolism of HCQ in the liver and genetic polymorphisms of metabolizing enzymes. In particular, single nucleotide polymorphisms may be important determinants of the differences in HCQ blood concentrations and adverse effects in different patients. Some CYP3A5, CYP2D6, and CYP2C8 genetic variants have been shown to affect the safety or efficacy of HCQ. The phenotypes predictive of ultra-rapid and poor metabolizers were considered high-risk phenotypes. After considering these high-risk phenotypes in different ethnic groups, it is predicted that a significant proportion of patients taking HCQ may be at risk for either therapeutic failure or severe toxicities [18–21]. Therefore, the prescribed dose of HCQ for patients with IgAN must not be based solely on the eGFR during the clinical treatment process. The initial therapeutic dose of HCQ can be determined based on the eGFR value [6, 22, 23]; however, during long-term follow-up, the HCQ blood concentration must be monitored closely and the prescribed dose must be adjusted accordingly. Hydroxychloroquine blood concentration and efficacy measurements reported in this study may provide a reference for HCQ dose adjustment in patients with IgAN.

The results of the current study suggest that HCQ effectively increases ALB and decreases LDL concentrations in patients with IgAN. The effect of HCQ on ALB may be due to its effects on proteinuria. The effect of HCQ on LDL has been reported in previous studies that demonstrated that HCQ is associated with lower LDL levels in patients with rheumatoid arthritis, improves lipid levels, and plays a role in preventing cardiovascular diseases [24, 25]. No serious adverse events were reported in the current study. One patient experienced mild renal impairment, which may be related to the short study period. Hydroxychloroquine has a retinal toxicity < 1% over five years and < 2% over 10 years at the recommended dose of 6.5 mg/kg [26]. In addition to retinopathy, adverse events of HCQ include cardiovascular side effects, skin complications, neurological and psychiatric complications, hepatic impairment, and gastrointestinal reactions [27]. Therefore, a longer observation period is required to determine the adverse events associated with HCQ in this patient population.

In conclusion, this single-center randomized controlled trial found that HCQ significantly and safely reduces proteinuria in patients with IgAN when combined with an optimal dose of RAASi. In addition, HCQ blood concentrations and efficacy were positively correlated in this study. The optimal HCQ blood concentration was 418.96 ng/mL at three months and 582.48 ng/mL at six months, allowing for individualized dose recommendations for HCQ for the management of IgAN.

This study is not without limitations. It is a single-center study with a small sample size and short follow-up period, which do not allow for further evaluation of the relationship between HCQ blood concentrations, long-term efficacy, and drug safety. Therefore, studies with larger sample sizes and longer observation periods are necessary.

## Supplementary Information

Below is the link to the electronic supplementary material.

Supplementary file1 Study flowchart (DOC 47 kb)

Supplementary file2 Patient clinical data. The proteinuria (a), ALB (b), LDL (c), and eGFR (d) of patients in the HCQ group are shown. *p< 0.05, **p< 0.01, ***p< 0.001. Abbreviations: HCQ, hydroxychloroquine; ALB, albumin; LDL, low-density lipoprotein cholesterol; eGFR, estimated glomerular filtration rate (DOC 1612 kb)

## Data Availability

Data available in the article or its supplementary materials.
