# Supplementary material for: Hydroxychloroquine blood concentrations and effects in Chinese patients with IgA nephropathy
Source: J Nephrol. 2024 Jul 24;37(8):2201–8. doi: 10.1007/s40620-024-02029-z (PMC11649793; doi:10.1007/s40620-024-02029-z)
Supplement: Supplementary file 1 — Supplementary file1 Study flowchart (DOC 47 kb) [file 40620_2024_2029_MOESM1_ESM.doc]

**Supplementary Table S1.** Baseline characteristics

|  | RAASi Group (n=35) | HCQ Group (n=38) | *p*-value |
| --- | --- | --- | --- |
| Age (years) | 39.34 ± 12.12 | 38.58 ± 10.39 | 0.4 |
| Sex (Male/Female) | 22/13 | 17/21 | 0.3 |
| Weight (kg) | 68.47 ± 16.85 | 68.84 ± 12.41 | 0.9 |
| BMI (kg/m^2^) | 24.35 ± 3.27 | 24.66 ± 3.42 | 0.6 |
| Systolic pressure (mmHg) | 131.52 ± 17.40 | 129.86 ± 18.03 | 0.3 |
| Diastolic pressure (mmHg) | 91.35 ± 13.07 | 90.71 ± 12.62 | 0.3 |
| 24-hour proteinuria (mg/d) | 832.00[701.00, 1395.00] | 942.50[685.00, 1725.92] | 0.6 |
| ALB (g/L) | 40.20 ± 3.16 | 39.43 ± 3.35 | 0.9 |
| BUN (mmol/L) | 5.18 ± 0.92 | 5.44 ± 1.60 | 0.4 |
| Scr (μmol/L) | 82.40 ± 25.17 | 77.08 ± 26.51 | 0.2 |
| TC (mmol/L) | 4.65 ± 0.86 | 4.89 ± 1.07 | 0.8 |
| TG (mmol/L) | 1.49 ± 0.71 | 1.93 ± 0.90 | 0.2 |
| LDL (mmol/L) | 2.76 ± 0.71 | 2.93 ± 0.92 | 0.9 |
| FBG (mmol/L) | 4.90 ± 0.51 | 4.62 ± 0.81 | 0.3 |
| Oxford histologic score |  |  |  |
| M 0/1 | 1/34 | 0/38 | 0.5 |
| E 0/1 | 33/2 | 38/0 | 0.2 |
| S 0/1 | 27/8 | 25/13 | 0.3 |
| T 0/1/2 | 30/4/1 | 29/8/1 | 0.7 |
| C 0/1/2 | 26/4/5 | 24/12/2 | 0.1 |
| Therapy with RAASi |  |  |  |
| ACEI | 2 (5.7%) | 4 (10.5%) |  |
| Perindopril | 1 (50%) | 3 (75%) |  |
| Benazepril | 1 (50%) | 1 (25%) |  |
| ARB | 32 (91.4%) | 32 (84%) |  |
| Valsartan | 16 (50%) | 19 (59%) |  |
| Irbesartan | 9 (28%) | 8 (25%) |  |
| Losartan | 7 (22%) | 5 (16%) |  |
| ACEI plus ARB | 1 (3%) | 2 (6%) |  |
| Perindopril plus Valsartan | 1 (100%) | 2 (100%) |  |
| Diabetes (%) | 3 (8.6%) | 2 (5.3%) | 0.7 |

Abbreviations: BMI, body mass index; ALB, albumin; BUN, blood urea nitrogen; Scr, serum creatinine; TC, total cholesterol; TG, triglycerides; LDL, low-density lipoprotein cholesterol; FBG, fasting blood glucose; RAASi, reninangiotensin-aldosterone system inhibitor; ACEI, angiotensin-converting enzyme inhibitor; ARB, angiotensin receptor blocker; M, mesangial hypercellularity (M0, <50% of glomeruli show mesangial hypercellularity; M1, >50% of glomeruli show mesangial hypercellularity); E, endocapillary hypercellularity (E0, no endocapillary hypercellularity; E1, any glomeruli show endocapillary hypercellularity); S, segmental glomerulosclerosis (S0, absent; S1, present in any glomeruli); T, tubular atrophy/interstitial fibrosis (T0, 0%-25% of cortical area; T1, 26%-50% of cortical area; T2, >50% of cortical area); C, crescents (C0: absent; C1, 0%-25% of glomeruli; C2, ≥25% of glomeruli).
