# Supplementary figures and images for: Hydroxychloroquine blood concentrations and effects in Chinese patients with IgA nephropathy
Source: J Nephrol. 2024 Jul 24;37(8):2201–8. doi: 10.1007/s40620-024-02029-z (PMC11649793; doi:10.1007/s40620-024-02029-z)

**Supplementary Figure S1.**

**
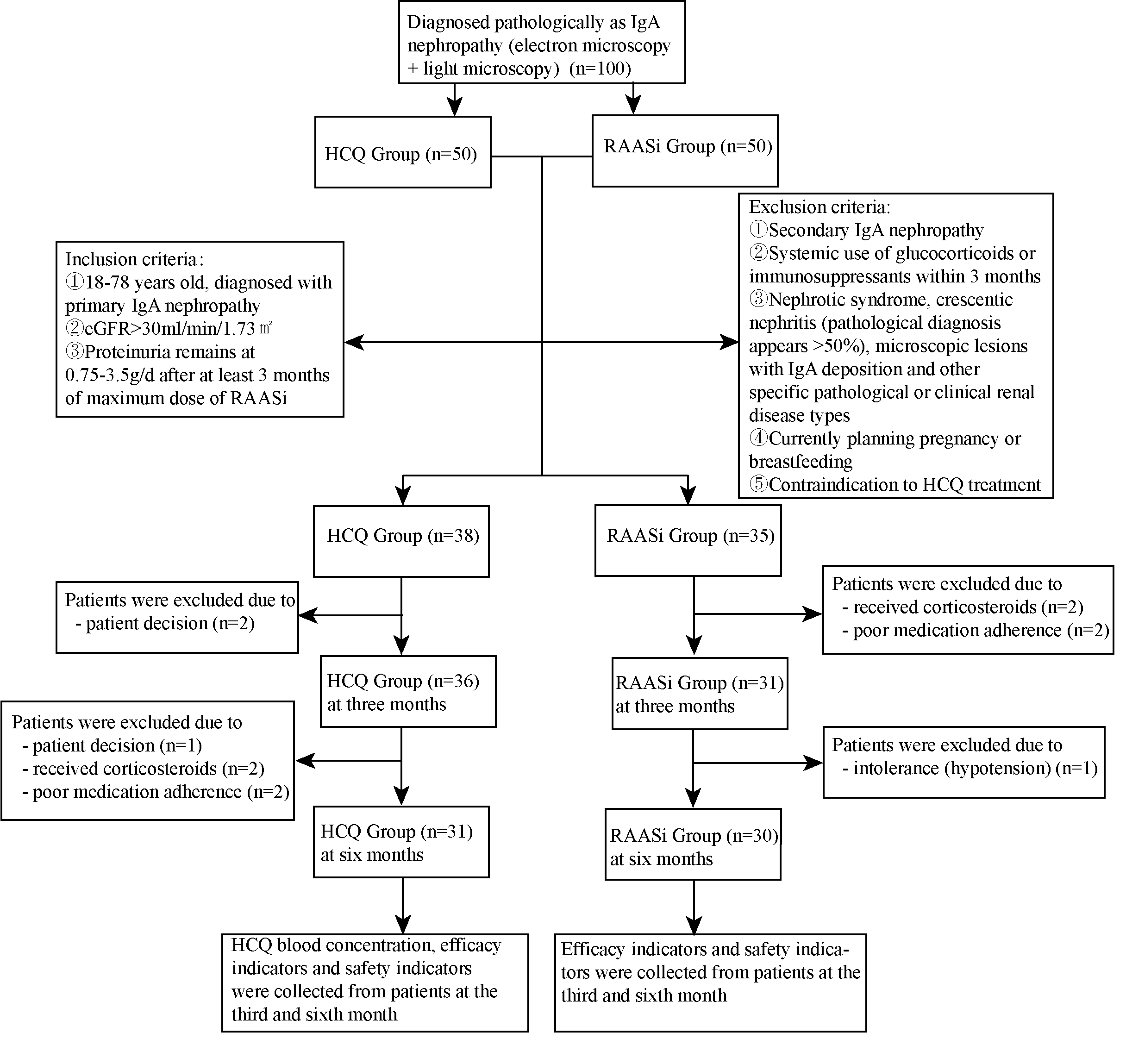
**

**Supplementary Figure S2.**


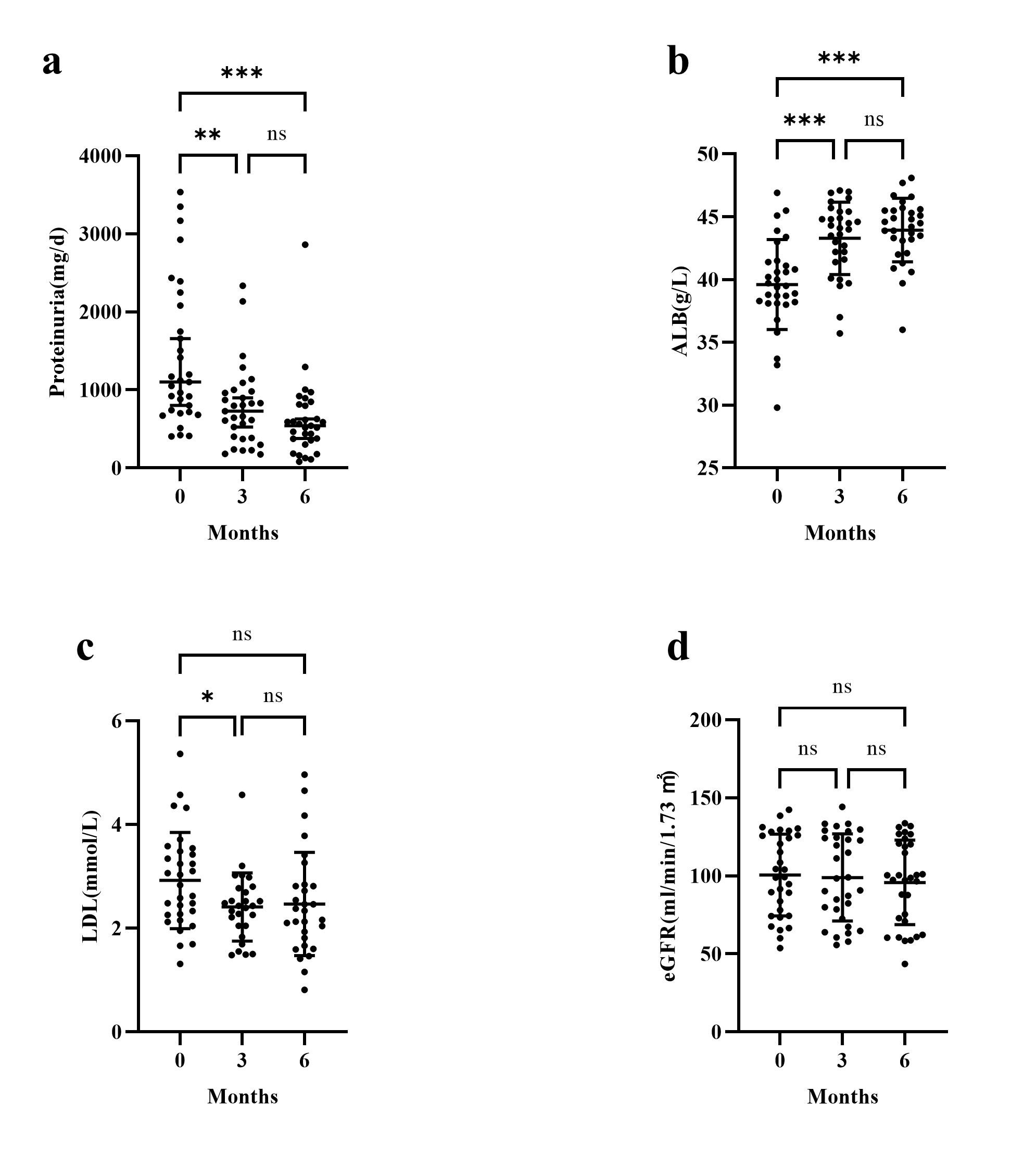

Supplement: Supplementary file 2 — Supplementary file2 Patient clinical data. The proteinuria (a), ALB (b), LDL (c), and eGFR (d) of patients in the HCQ group are shown. *p< 0.05, **p< 0.01, ***p< 0.001. Abbreviations: HCQ, hydroxychloroquine; ALB, albumin; LDL, low-density lipoprotein cholesterol; eGFR, estimated glomerular filtration rate (DOC 1612 kb) [file 40620_2024_2029_MOESM2_ESM.doc]
